# The Determinants of User Acceptance of Mobile Medical Platforms: An Investigation Integrating the TPB, TAM, and Patient-Centered Factors

**DOI:** 10.3390/ijerph191710758

**Published:** 2022-08-29

**Authors:** Hailiang Wang, Jiaxin Zhang, Yan Luximon, Mingfu Qin, Ping Geng, Da Tao

**Affiliations:** 1 School of Design, The Hong Kong Polytechnic University, Hong Kong, China; 2 School of Primary Education, Hunan Vocational College for Nationalities, Yueyang 414000, China; 3 Department of Applied Biology and Chemical Technology, The Hong Kong Polytechnic University, Hong Kong, China; 4 Institute of Human Factors and Ergonomics, College of Mechatronics and Control Engineering, Shenzhen University, Shenzhen 518060, China

**Keywords:** technology acceptance, mobile medical platform, TPB, TAM, patient-centered factors

## Abstract

Mobile medical platforms (MMPs) can make medical services more accessible and effective. However, the patient-centered factors that influence patients’ acceptance of MMPs are not well understood. Our study examined the factors affecting patients’ acceptance of MMPs by integrating the theory of planned behavior (TPB), the technology acceptance model (TAM), and three patient-centered factors (i.e., perceived convenience, perceived credibility, and perceived privacy risk). Three hundred and eighty-nine Chinese respondents were recruited in this study and completed a self-administered online questionnaire that included items adapted from validated measurement scales. The partial least squares structural equation modeling results revealed that perceived privacy risk, perceived credibility, and perceived ease of use directly determined the perceived usefulness of an MMP. Perceived convenience, perceived credibility, and perceived usefulness significantly affected the patients’ attitudes toward MMPs. Perceived usefulness, attitude, perceived privacy risk, and perceived behavioral control were important determinants of the patients’ behavioral intentions to use MMPs. Behavioral intention and perceived behavioral control significantly influenced perceived effective use. Perceived credibility and perceived ease of use significantly affected perceived convenience. However, social influence had no significant effect on attitude and behavioral intention. The study provides important theoretical and practical implications, which could help practitioners enhance the patients’ use of MMPs for their healthcare activities.

## 1. Introduction

The increasing prevalence of chronic diseases and suboptimal health conditions is one of the most serious issues facing public healthcare worldwide. The statistics show that around one in three adults suffers from more than one chronic condition [1]. In China, the prevalence rate of chronic diseases (e.g., hypertension, diabetes, and hypercholesterolemia) among adults above the age of 60 is as high as 75.8% [2]. With such a large population suffering from health problems, the demands for medical services have exceeded the supply that can be offered by healthcare systems. As traditional medical services become overstrained, they can become costly and inaccessible for a large portion of patients [3].

One potential solution for meeting the demands for medical services under such circumstances is the use of mobile medical platforms (MMPs), which can make medical services and health management more accessible. MMPs offer a wide range of functions that allow patients to make medical appointments, consult professional physicians, purchase medicines, acquire health information, and even undergo medical examinations via the complementary use of wearable devices [4]. MMPs have many advantages over the traditional delivery of medical services. For example, the use of MMPs saves the time that patients would otherwise use to wait for offline clinical appointments in conventional medical services [5]. The patients can also easily contact specific medical professionals and seek medical advice remotely through MMPs [6,7,8]. Additionally, MMPs can facilitate communication between patients, physicians, and clinics/hospitals with various technology-assisted tools that are provided in the platforms, such as electronic notifications, messaging, video chat, comments, and dashboards. Health education can also be delivered to the public through MMPs [9].

Despite the many benefits of MMPs, they are not always sustainably used and accepted by their potential users. A recent healthcare app survey [10] reported that on average only 44% of users showed a 30-day retention with the apps and the proportion decreased to 36% for a 90-day retention. Similarly, a recent literature review on users’ engagement and retention of mobile health apps [11] showed that retaining the users of MMPs was really challenging and many barriers (e.g., usefulness) were yet to be addressed for retention and long-term engagement. Therefore, the troubling problem of underused or unaccepted MMPs remains an important concern in mobile health practice. In addition, knowledge of the factors influencing the use of such services remains incomplete. While a number of factors, such as perceived ease of use, perceived usefulness, attitude, social cue design, subjective norms, and perceived behavioral control, were examined in relation to the user acceptance of MMPs [7,12,13], little research has focused on the patient-centered factors that should be well addressed to provide patient-centered services in MMPs. Several of the important patient-centered factors include perceived convenience, perceived credibility, and perceived privacy risk. The patient-centered services are designed to foster relationships between patients and medical service providers in the context of healthcare, specifically by facilitating communication between patients and medical service providers, and reinforcing their mutual trust [14]. Unlike traditional medical services, medical services on MMPs are delivered online, and patients are unable to meet their medical service providers in person, which makes patient-centered services less available. Thus, it is important to understand patient-centered factors that influence perceived communication quality and trust by patients using MMPs.

In the present study, we extend the literature by investigating how the acceptance of MMPs is shaped by the provision of patient-centered services. We proposed that patient-centered MMPs could offer high-quality communication and trust, and user acceptance can be explained by combining multiple constructs from the technology acceptance model (TAM) and the theory of planned behavior (TPB) with three patient-centered factors (i.e., perceived convenience, perceived credibility, and perceived privacy risk). To investigate the determinants of user acceptance of MMPs and identify the roles of patient-centered factors that affect the acceptance of MMPs, we proposed and examined a theoretical model via partial least squares structural equation modeling (PLS-SEM), with data collected from an empirical study. Overall, our study aimed to provide insights into the factors influencing the quality of patient-centered services on MMPs and to identify ways in which various stakeholders can improve user acceptance of MMPs.

## 2. Theoretical Background and Research Hypotheses

### 2.1. Theoretical Background

A review of the literature indicates that many theories existed to explain technology acceptance and usage behaviors, such as TPB, TAM, Theory of Reasoned Action (TRA), Social Cognitive Theory (SCT), Motivational Model (MM), and Innovation Diffusion Theory (IDT). While many of the theories (e.g., TRA, SCT and MM) focus more on the psychological side of individuals’ behaviors, TAM enables easy integration with contextual factors that are more likely to lead to practical implications in technology design. In addition, previous reviews showed that TAM was usually able to explain as high as 50% of IT acceptance behaviors and thus was identified by its robustness, parsimony, and predictive power in explaining technology acceptance over a wide range of technology scenarios [15,16,17]. In the present study, we also chose TAM as the theoretical framework.

The TAM predicts and explains a user’s acceptance of information technology. It emphasizes the important role of perceived usefulness and perceived ease of use of a given technology in affecting user attitudes and behavioral intention to use the technology [18]. However, the TAM also accounts for the influence of external variables on behavioral intention, which ultimately depends on the research context [19]. The TAM was applied in various information technologies [9,20,21]. For example, Zhang and Luximon [7] applied the TAM to investigate the relationships between the determinants of patient trust and technology acceptance. Li [22] employed the TAM to examine users’ acceptance of mHealth applications, and explored the effects of external variables, such as perceived interactivity, perceived personalization, privacy concerns, and trust. In addition, Deng et al. [13] integrated TAM constructs with perceived risk and trust to predict users’ behavioral intention toward mobile health services.

Nonetheless, in emphasizing the role of technological factors (e.g., perceived ease of use, perceived usefulness, perceived interactivity) in influencing users’ behavioral intention to adopt technologies, most of the TAM studies have neglected the influence of users’ abilities and resources as well as social environmental factors (e.g., social norms) [23]. Here, in order to construct a comprehensive model, we integrated the TAM with the TPB to build the theoretical foundation for our research. The TPB explains an individual’s behavioral intention from the perspective of cognitive self-regulation [24]. Specifically, the TPB posits that behavioral intention depends on an individual’s ability to perform the specific behavior (i.e., perceived behavioral control), the level of social approval that the individual perceives in undertaking the specific behavior (i.e., social influence), and attitudes toward the specific behavior [24,25]. The TPB has been widely used to interpret health-related behaviors. For instance, Bao et al. extended the TPB by adding the constructs of trust and risk and used their model to predict patients’ use of online health information [26]. In addition, Deng et al. used the TPB to compare the predictors of behavioral intention toward mobile health services in middle-aged and older adults [27].

To explore how MMPs can provide patient-centered services, we took the constructs from the TAM and TPB as the basis for a patient’s behavioral intention to adopt an MMP, and proposed adding perceived convenience, perceived credibility, and perceived privacy risk as external patient-centered factors in our model. Specifically, a patient’s perceived ease of use and perceived usefulness are associated with the usability dimension [8,28,29], which serves as the basis of patient-centered services in MMPs [30]. In addition, the perceived convenience and perceived credibility may enhance the patient-centered services of MMPs. Indeed, perceived convenience reflects the effectiveness of communication between patients and physicians enabled by MMPs [31], while perceived credibility reflects the quality of the information provided by MMPs [32]. Hence, both of the factors critically influence the quality of communication in medical services. However, perceived privacy risk can hinder the development of patient-centered services. As the patients using MMPs have to disclose sensitive information [8,13], they may be reluctant to adopt MMPs if they perceive a high level of privacy risk [7,8,13,33]. A patient’s perceived behavioral control can influence the relationship between their ability and resources and their acceptance of an MMP [26]. Social influence, which refers to the influence of the social environment, was shown to determine the acceptance of various information technologies [26,34,35], and may also determine the acceptance of MMPs. In addition, we used perceived effective use to evaluate the extent to which patients’ behavioral intention increases their reliance on using an MMP to address health problems and perform health management. The proposed hypotheses and research model are presented in the following sections.

### 2.2. Research Hypotheses

**Figure 1:**
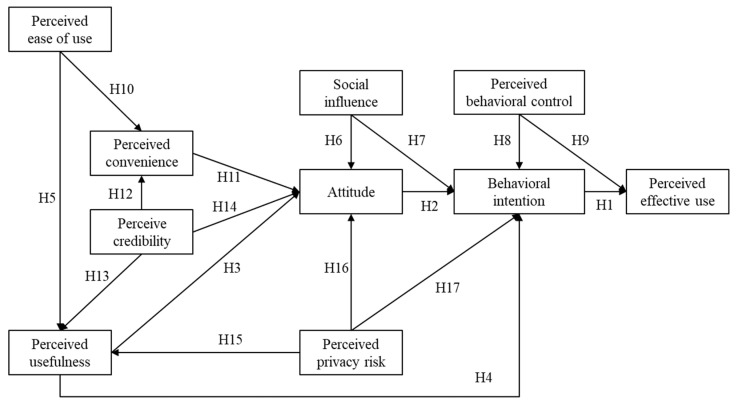
The proposed MMP acceptance model.

Based on a literature review of the theoretical background, we proposed 17 hypotheses and developed a theoretical MMP acceptance accordingly (Figure 1).

#### 2.2.1. Behavioral Intention, Attitude, and Perceived Effective Use

Behavioral intention refers to the extent to which an individual will use a technology [36]. It was widely recognized as a predictor of an individual’s actual acceptance behavior [36,37,38,39,40,41,42,43]. Accordingly, we used behavioral intention as the agent of acceptance in our model. We also further explored the relationship between a patient’s behavioral intention toward an MMP and their reliance on that MMP by measuring their level of perceived effective use [44]. We expected that the patients with high behavioral intention to use MMPs will be prone to relying on MMPs and therefore are likely to adopt MMPs for their health management. Thus, we proposed the following hypothesis:

**H1.**  *Behavioral intention positively affects perceived effective use.*

In our study, attitude refers to the degree of negative or positive feelings that an individual has toward technology use. The studies have consistently shown that an individual with a positive attitude toward a given technology will have a strong intention to use that technology [40,45,46]. In the original TAM, the link between attitude and behavioral intention was shown to be the most stable [45,47]. Thus, the following hypothesis was proposed:

**H2.**  *Attitude positively affects behavioral intention to use an MMP.*

#### 2.2.2. Perceived Usefulness, Perceived Ease of Use, and Attitude

Based on the definition of Davis [18], we defined perceived usefulness as the extent to which an individual believes that using an MMP will enhance their healthcare management. The MMPs are designed to offer users better access to healthcare services, such as making appointments, receiving remote diagnoses from doctors, and accessing health-related information. The studies consistently showed that perceived usefulness significantly influences an individual’s attitude and behavioral intention to use a technology [29,34,39,40,43]. In line with the definition proposed by Davis [18], we defined perceived ease of use as the extent to which an individual believes that using an MMP will be an effortless endeavor. Given that MMPs are smartphone-based applications, we expected that the extent to which patients perceive their use of such applications as effortless will influence their perception of the benefits that MMPs provide. The perceived ease of use was shown to be a primary determinant of perceived usefulness [36,42,43]. Thus, we hypothesized the following:

**H3.**  *Perceived usefulness positively affects a patient’s attitude to use an MMP.*

**H4.**  *Perceived usefulness positively affects a patient’s behavioral intention to use an MMP.*

**H5.**  *Perceived ease of use positively affects perceived usefulness.*

#### 2.2.3. Social Influence

Social influence refers to the degree to which an individual’s behavior (e.g., using an MMP for healthcare management) is influenced by the attitudes of others (e.g., family members and/or friends). People may change their behaviors based on suggestions from important family members or friends to strengthen these social relationships [34,48]. Numerous studies, e.g., [17,34,49], reported that social influence is a significant determinant of attitude and behavioral intention. Thus, we proposed the following hypotheses:

**H6.**  *Social influence positively affects a patient’s attitude.*

**H7.**  *Social influence positively affects a patient’s behavioral intention to use an MMP.*

#### 2.2.4. Perceived Behavioral Control

Perceived behavioral control refers to the degree to which an individual will perceive the presence or absence of the resources needed to perform a given behavior (e.g., using an MMP for healthcare management). The studies found that perceived behavioral control is a significant determinant of behavioral intention [36,50,51]. A person with higher perceived behavioral control may be more likely to use a given technology effectively. Therefore, we hypothesized the following:

**H8.**  *Perceived behavioral control positively affects a patient’s behavioral intention to use an MMP.*

**H9.**  *Perceived behavioral control positively affects a patient’s perceived effective use of an MMP.*

#### 2.2.5. Perceived Convenience

Convenience is associated with the benefits of time and place and was studied as a critical determinant of technology acceptance in m-commerce [31,52,53]. The convenience of healthcare services is an issue that has received increasing attention in recent years, as mobile technologies today allow patients to access their health information and medical services in an effective and timely manner [31]. In the context of MMPs, perceived convenience refers to the degree to which an individual perceives that they can access health information/services anytime and anywhere. The studies reported that perceived convenience is an important antecedent of attitude and that perceived ease of use can positively affect perceived convenience [52]. As there is little information on the effect of perceived convenience on behavioral intention in MMPs, we proposed the following hypotheses:

**H10.**  *Perceived ease of use positively affects perceived convenience.*

**H11.**  *Perceived convenience positively affects a patient’s attitude toward the use of an MMP.*

#### 2.2.6. Perceived Credibility

Perceived credibility refers to the degree to which an individual perceives information and services provided by a given technology to be reliable [40,54]. In the context of MMPs, the perceived credibility reflects the degree to which a patient believes the information provided by an MMP. The studies demonstrated that information credibility is important for the success of the health-related portals used for health management [40]. In particular, when it comes to health information, the more credible the information, the more useful it is perceived [40,55,56]. When patients perceive a high level of credibility in health information, they are more likely to perceive the platform as convenient. Therefore, we proposed the following hypotheses:

**H12.**  *Perceived credibility positively affects the perceived convenience of an MMP.*

**H13.**  *Perceived credibility positively affects the perceived usefulness of an MMP.*

**H14.**  *Perceived credibility positively affects a patient’s attitude toward an MMP.*

#### 2.2.7. Perceived Privacy Risk

The perceived privacy risk refers to the degree to which an individual perceives that an MMP may steal their personal information [57]. MMPs collect sensitive information from their users, such as their health and personal data. Increasingly, consumers are raising privacy concerns about vague and unfair data collection and privacy policies [57]. Such concerns are further reinforced by the secondary use of users’ health and personal data [33,58,59]. Faced with a high privacy risk, patients may develop negative attitudes toward MMPs [58,60]. MMP users may also face a tradeoff between perceived usefulness and perceived privacy risk [33,61]. Thus, we hypothesized the following:

**H15.**  *Perceived privacy risk negatively affects the perceived usefulness of an MMP.*

**H16.**  *Perceived privacy risk negatively affects a patient’s attitude toward an MMP.*

**H17.**  *Perceived privacy risk negatively affects a patient’s behavioral intention to use an MMP.*

## 3. Research Methodology

### 3.1. Participants

A convenience sampling method was applied and the data were collected via a professional web-based survey platform (https://www.sojump.com), which was widely used in previous studies [34,43]. Individuals who owned a smartphone and had experience of using MMPs were eligible to participate in the study. Four hundred and fifty questionnaires were randomly distributed, and 389 valid responses were received (response rate: 86.4%) and used for data analysis (Table 1). The study was approved by the Institutional Review Board of Shenzhen University. In addition, all of the participants were informed that none of their medical information would be collected.

### 3.2. Instruments

We designed a questionnaire by adopting validated scales that were identified through an extensive literature review of the studies on technology acceptance. We modified several measurement items to make them suitable for the context of MMPs. The questionnaire included three sections. The first section provided a brief description of MMPs and associated examples that were already implemented in the healthcare industry, the second section asked the participants for their demographic information (e.g., age, gender, Internet use, and e-Health use experience), while the third section included items that measured the constructs in our proposed research model. The participants were instructed to answer items in the third section based on their experience with one of the typical MMPs that they recently or frequently used. The construct items were rated with 7-point Likert-type scales, ranging from 1 “strongly disagree” to 7 “strongly agree”. Table 2 presents the measurement items and sources of the constructs. 

### 3.3. Data Analysis

We used PLS-SEM to verify the measurement model and examine the structural model [65]. All of the analyses were performed using SmartPLS 3.0 (SmartPLS GmbH, Boenningstedt, Germany) [66]. To verify the measurement model, we evaluated the model’s internal consistency reliability, convergent validity, and discriminant validity. Specifically, good reliability is achieved if Cronbach’s alpha is greater than 0.7 [67]. The convergent validity is deemed acceptable when: (1) the factor loading of each construct is greater than 0.7; (2) composite reliability and the average variance extracted (AVE) for each construct are greater than 0.7 and 0.5, respectively [68,69,70]. The discriminant validity is deemed acceptable when: (1) the outer loading of each construct is greater than any of its cross-loadings on other constructs; and (2) the square root of the AVE for each construct is greater than its correlations with any other constructs [68,70,71]. We examined the structural model by calculating the path coefficients and determining their statistical significance by running 5000 bootstrap subsamples. We also calculated the R^2^ values, which indicate the amount of variance explained by the independent variables.

## 4. Results

### 4.1. Descriptive Statistics

Table 3 summarizes the participants’ responses to measurement items for the constructs in our model. The average ratings for the measurement items ranged from 4.33 (PPR1) to 5.60 (PU2).

### 4.2. Assessment of the Measurement Model 

As shown in Table 4, all of the Cronbach’s alpha values were greater than 0.7, indicating that the constructs had good reliability. In addition, the factor loadings for all of the items were greater than 0.7, all of the AVEs exceeded 0.5, and all of the composite reliability values were greater than 0.7, indicating that the constructs had good convergent validity.

Table 5 shows that the square root of the AVE for each construct was greater than its correlation coefficients with the other constructs. Table 6 shows that each item had a higher factor loading on its corresponding construct than its cross-loadings on the other factors. Together, these findings indicated that the measurement model had satisfactory discriminant validity.

### 4.3. Structural Model Testing

**Figure 2:**
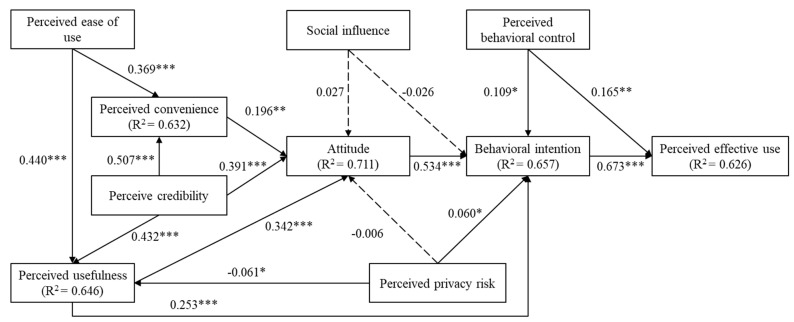
The results of the structural model (note: * *p* < 0.05; ** *p* < 0.01; *** *p* < 0.001).

The results of hypothesis testing are summarized in Table 7 and illustrated in Figure 2. The perceived effective use was predicted by perceived behavioral control (β = 0.165, *p* < 0.01) and behavioral intention (β = 0.673, *p* < 0.001), while social influence had no significant effect on behavioral intention. Therefore, H1 and H9 were supported, but H7 was rejected. Attitude (β = 0.534, *p* < 0.001), perceived behavioral control (β = 0.109, *p* < 0.05), and perceived privacy risk (β = 0.06, *p* < 0.05) had direct effects on the behavioral intention, supporting H2, H8, and H17, respectively. Perceived usefulness (β = 0.342, *p* < 0.001), perceived convenience (β = 0.196, *p* < 0.01), and perceived credibility (β = 0.391, *p* < 0.001) positively affected attitude, while social influence and perceived privacy risk had no significant effect on attitude. Thus, H3, H11, and H14 were supported, but H6 and H16 were rejected. Perceived ease of use (β = 0.369, *p* < 0.001) and perceived credibility (β = 0.507, *p* < 0.001) had positive effects on perceived convenience, supporting H10 and H12. Perceived ease of use (β = 0.440, *p* < 0.001), perceived credibility (β = 0.432, *p* < 0.001), and perceived privacy risk (β = 0.061, *p* < 0.05) had significant effects on perceived usefulness. Therefore, H5, H13, and H15 were supported. Overall, the results indicated that our research model explained 62.6% of the variance in perceived effective use, 65.7% of the variance in behavioral intention, 71.1% of the variance in attitude, 63.2% of the variance in perceived convenience, and 64.6% of the variance in perceived usefulness.

## 5. Discussion

In this study, we constructed a research model to investigate several patient-centered factors that can predict patients’ acceptance of MMPs. Based on the TAM and the TPB, we examined the effects of perceived convenience, perceived credibility, perceived privacy risk, social influence, perceived behavioral control, and attitude on patients’ behavioral intention to use MMPs, and tested whether perceived behavioral control and behavioral intention influenced perceived effective use.

### 5.1. Primary Findings

The results revealed that the patient-centered factors affected the patients’ acceptance of MMPs. Specifically, we found that perceived credibility and perceived convenience were critical patient-centered factors that positively affected the acceptance of MMPs. This finding is consistent with those of previous studies [31,32]. As perceived credibility reflects the quality of the information provided by MMPs and perceived convenience relates to the benefits of time and effectiveness that patients derive from using MMPs, it is reasonable that the two factors positively influence patients’ perception and attitude toward MMP services. However, perceived credibility had a stronger influence on patients’ acceptance of MMPs than perceived convenience. One reason for this may be that perceived credibility can not only affect a patient’s attitude directly but also through perceived usefulness and perceived convenience. In contrast, perceived convenience only influenced patients’ attitude directly. Another reason may be that the path coefficient between perceived credibility and attitude (β = 0.391, *p* < 0.001) was greater than that between perceived convenience and attitude (β = 0.196, *p* < 0.001). We therefore deduced that the stronger effect of perceived credibility (relative to perceived convenience) was due to the fundamental role of trust conveyed by perceived credibility. The studies showed that trust is vital to the acceptance of online medical services [8,72,73]. Trust is also important for building relationships between the patients, physicians, and medical platforms [23]. If the patients can trust MMPs, they are more likely to perceive the information provided by MMPs as believable. Although convenience may be an important motivator in a patient’s decision to adopt an MMP, the patients are likely to prioritize the trust and credibility of the medical information received when deciding whether or not to use MMPs to manage their health problems. This explains why perceived credibility had a stronger effect than perceived convenience on MMP acceptance.

Consistent with the previous studies, perceived privacy risk negatively affected perceived usefulness and behavioral intention to use MMPs [33,74]. However, the path coefficients between perceived privacy risk and perceived usefulness (β = −0.061, *p* < 0.05), and between perceived privacy risk and behavioral intention (β = −0.060, *p* < 0.05) were small. Furthermore, perceived privacy risk did not affect the patients’ attitudes toward MMPs. The privacy calculus theory holds that users tend to compare social benefits with risk when disclosing their personal information [75]. This study revealed that the benefits of MMPs (e.g., perceived usefulness and perceived convenience) played a more important role than perceived privacy risk in shaping the patients’ attitudes toward MMPs. This result also suggests that the potential risk of losing one’s sensitive information via the use of an MMP may not be a serious cause for concern when it comes to adopting that MMP. However, as perceived privacy risk can negatively affect the perceived usefulness and attitude, efforts should be made to reduce the perceived privacy risk associated with the use of MMPs.

This study also confirmed the effects of the TAM and TPB constructs on patient-centered factors and patients’ intention to adopt MMPs. For the TAM constructs, perceived ease of use was positively associated with perceived convenience (β = 0.369, *p* < 0.001) and perceived usefulness (β = 0.440, *p* < 0.001). This indicates that reducing the complexity of the user interface and allowing users to operate MMPs effortlessly are the antecedents for the development of convenient and useful MMPs. We also found that perceived usefulness was a key predictor of attitude (β = 0.324, *p* < 0.001) and behavioral intention (β = 0.253, *p* < 0.001) toward MMPs. This indicates that patients are more likely to adopt MMPs if they perceive MMPs to have the capacity to improve their health management. Our results also consistently confirmed the effects of perceived usefulness in MMPs, which were previously documented in the studies of health information acceptance [22].

Regarding the TPB constructs, although social influence was found to predict the acceptance of many information technologies [35,76,77], our results showed that it did not affect the patients’ attitudes and behavioral intentions toward MMPs. In other words, the attitudes of patients’ families or friends did not influence their intention to adopt MMPs. The reason for this finding may be that MMPs in China remain in the initial phase of implementation and are not yet used widely. Accordingly, as social influence had little effect on acceptance intention in our study, the participants were less likely to share and recommend MMPs to others. However, we found that perceived behavioral control had a positive impact on behavioral intention (β = 0.109, *p* < 0.05) and perceived effective use (β = 0.165, *p* < 0.01). This is in line with previous research, which linked behavioral control to behavioral intention and perceived effective use [51]. We also found that perceived behavioral control affected the acceptance of MMPs, indicating that patients’ personal abilities and resources influenced their decisions to use MMPs. Thus, future studies could explore how different types of personal abilities influence the acceptance of MMPs. Additionally, our findings confirmed that behavioral intention strongly affected the perceived effective use of MMPs. In line with previous research linking perceived effective use to patients’ reliance on MMPs [44], our findings imply that patients who have a stronger intention to adopt MMPs will also use MMPs to address their health problems and manage their health.

### 5.2. Theoretical and Practical Implications

In this study, we built a theoretical model to explore the determinants of behavioral intention to use MMPs from a patient-centered perspective. The findings revealed that perceived credibility, perceived convenience, and perceived privacy risk affected patients’ attitudes toward MMPs, and therefore provided new insights for improving the quality of communication and trust in MMPs. Specifically, our model highlighted the importance of perceived credibility, as this construct not only affected patients’ attitude directly but also through perceived convenience and perceived usefulness. As perceived credibility is associated with the quality of the information provided by MMPs, the results suggest that further research is needed to identify how high-quality information can be provided in MMPs. In contrast, the perceived privacy risk may be less likely to affect patients’ behavioral intention to use MMPs. This may imply that privacy risk is not the main concern of patients deciding to adopt MMPs. However, we recommend that future studies explore the mediators between perceived privacy risk and behavioral intention, as the use of MMPs involves transactions with and storage of sensitive information. As our findings showed, the perceived ease of use and perceived usefulness were vital determinants of the acceptance of MMPs. This suggested that the usability dimension remained the foundation of patient-centered services in MMPs. Additionally, we found that perceived behavioral control affected behavioral intention to use MMPs. Future studies could thus explore ways to improve the usability of MMPs with respect to patients with different abilities and resources.

Practically, our findings have several implications for the relevant MMP stakeholders. First, the quality of information provided by MMPs should be prioritized to enhance their patient-centered services. Our findings regarding perceived credibility indicated that providing accurate, up-to-date, believable, and authoritative medical information is vital to the acceptance of MMPs. Thus, practitioners should make concerted efforts to improve the credibility of the information provided in MMPs. Second, our findings underscored the importance of a patient-centered user interface. Our research found a strong relationship between behavioral intention and perceived effective use. Specifically, the patients are likely to continuously use MMPs to manage their health if their behavioral intention to use MMPs was formed. To increase the actual use of MMPs and reduce the strain on traditional medical services, practitioners should actively promote the acceptance of MMPs.

### 5.3. Limitations and Future Work

Despite the implications discussed above, this study has several limitations. First, the survey was only conducted in a Chinese population. As cultural differences can affect social norms and users’ perceptions and attitudes, validation of the findings in other cultural regions will be required prior to their application [77]. We also recommend a cross-cultural comparison of the determinants of MMP acceptance. Second, as MMPs are currently in their initial phase in China, the findings may only be applicable to predict patients’ behaviors during this phase. Further studies on the determinants of acceptance in the post-acceptance phase should be conducted. Third, as with many of the previous studies [22,34,43,78,79], the survey in this study was not based on a real usage scenario, which means that the participants did not really use MMPs for healthcare activities before they answered the questionnaire items. Instead, the participants were instructed to answer the questionnaire items based on their previous experience with the MMPs that they recently or frequently used. This might lead to some bias for accurate data elicitation due to perceptions that rely heavily on fresh memory (e.g., perceived credibility). Thus, future studies could extend our research model in real usage scenarios to reflect more accurate user perceptions.

## 6. Conclusions

Our study investigated how patient-centered factors influence the acceptance of MMPs and verified a theoretical acceptance model by integrating the TAM and the TPB with three patient-centered factors (i.e., perceived credibility, perceived convenience, and perceived privacy risk). Our integrated model explained 65.7% of the variance in behavioral intention to adopt MMPs, and 14 of the 17 proposed hypotheses were supported. Specifically, the patient-centered factors had various effects on their attitudes and behavioral intention to use MMPs. In addition, behavioral intention had a strong effect on perceived effective use. Future studies could extend our study by examining different MMPs during the post-implementation stage.

## Figures and Tables

**Table 1:** Demographic characteristics of the participants (*n* = 389).

Items	Type	Number of Participants	Percentage (%)
Gender	Male	150	38.6%
	Female	239	61.4%
Age	<18	27	6.9%
	18–30	224	57.6%
	31–40	108	27.8%
	41–50	24	6.2%
	51 or above	6	1.5%
Education	High school or lower	8	2.1%
	College	90	23.1%
	Bachelor’s degree	270	69.4%
	Master’s degree or above	21	5.4%
Usage of smartphone (hours/day)	<1	3	0.8%
	1–4	135	34.7%
	5–8	192	49.4%
	>8	59	15.2%
Usage of mobile medical platforms	More than once/day	14	3.6%
	Once/day	54	13.9%
	Once/week	151	38.8%
	Once/month	126	32.4%
	Once/6 months	44	11.3%

**Table 2:** Measurement items for the constructs in the research model.

Constructs	Items
Perceived ease of use (PEOU) [19]	PEOU1 Learning to use MMPs is easy for me.
	PEOU2 I find it easy to get MMPs to do what I want them to do.
	PEOU3 It is easy for me to become skillful at using MMPs.
	PEOU4 I find MMPs easy to use.
Perceived usefulness (PU) [19]	PU1 Using MMPs improves my ability of health management.
	PU2 Using MMPs helps me save time in managing my health.
	PU3 Using MMPs enhances the effectiveness of my health management.
	PU4 I find MMPs to be useful in my health management.
Attitude (ATT) [19]	ATT1 Using MMPs is a good idea.
	ATT2 Using MMPs is a wise idea.
	ATT3 I like the idea of using MMPs.
Social influence (SI) [25]	SI1 My family members influence my decision to use MMPs.
	SI2 My friends influence my decision to use MMPs.
Perceived behavioral control (PBC) [62]	PBC1 I have the ability to use MMPs to manage my health.
	PBC2 I have the resources (including training opportunity) that allow me to use MMPs for my health management.
Perceived convenience (PCV) [63]	PCV1 I can access health care services at any time via MMPs.
	PCV2 I can access health care services at any place via MMPs.
	PCV3 MMPs are a convenient way for me to access health care services.
Perceived privacy risks (PPR) [25]	PPR1 I am concerned that MMPs collect too much personal information from me.
	PPR2 I am concerned that MMPs will share my personal information with other entities without my authorization.
Perceived credibility (PCB) [64]	PCB1 The information provided by MMPs is up-to-date.
	PCB2 The information provided by MMPs is accurate.
	PCB3 The information provided by MMPs is trustworthy.
	PCB4 The information provided by MMPs is authoritative.
Behavioral intention (BI) [36]	BI1 I intent to use this MMP when I need it in the future.
	BI2 I predict that I will use the mobile medical service in the future.
	BI3 I plan to use MMPs in the future.
Perceived effective use (PEU) [44]	To what extent do you use MMPs as much as you should use it?

**Table 3:** Descriptive statistics of the measurement items for the constructs.

Constructs	Items	Mean	SD	95%CI
Attitude (ATT)	ATT1	5.46	0.88	[5.37, 5.55]
	ATT2	5.40	0.92	[5.31, 5.49]
	ATT3	5.38	0.96	[5.28, 5.47]
Behavioral intention (BI)	BI1	5.53	0.91	[5.44, 5.63]
	BI2	5.57	0.93	[5.48, 5.67]
	BI3	5.55	0.94	[5.46, 5.65]
Perceived credibility (PCB)	PCB1	5.20	0.92	[5.10, 5.29]
	PCB2	5.04	0.91	[4.95, 5.13]
	PCB3	5.16	0.93	[5.07, 5.26]
	PCB4	4.95	0.98	[4.85, 5.04]
Perceived convenience (PCV)	PCV1	5.35	0.89	[5.26, 5.44]
	PCV2	5.32	0.99	[5.22, 5.42]
	PCV3	5.52	0.96	[5.42, 5.61]
Perceived ease of use (PEOU)	PEOU1	5.53	0.90	[5.44, 5.62]
	PEOU2	5.39	0.96	[5.29, 5.48]
	PEOU3	5.52	0.96	[5.43, 5.62]
	PEOU4	5.55	0.97	[5.45, 5.64]
Perceived privacy risks (PPR)	PPR1	4.33	1.42	[4.19, 4.47]
	PPR2	4.46	1.61	[4.30, 4.62]
Perceived behavioral control (PBC)	PBC1	5.35	0.95	[5.26, 5.45]
	PBC2	4.92	1.18	[4.80, 5.04]
Perceived usefulness (PU)	PU1	5.35	0.97	[5.25, 5.44]
	PU2	5.60	1.00	[5.50, 5.70]
	PU3	5.38	0.98	[5.28, 5.48]
	PU4	5.47	0.93	[5.38, 5.57]
Social influence (SI)	SI1	4.56	1.27	[4.43, 4.68]
	SI2	4.60	1.23	[4.48, 4.72]
Perceived effective use (PEU)	PEU	5.45	0.83	[5.36, 5.53]

**Table 4:** Reliability and convergent validity of the constructs.

Constructs	Items	Item Loadings	*t*-Values	AVEs	Composite Reliability	Cronbach’s Alpha
Attitude (ATT)	ATT1	0.892	65.155	0.795	0.921	0.871
	ATT2	0.896	69.758			
	ATT3	0.887	62.023			
Behavioral intention (BI)	BI1	0.919	85.643	0.840	0.940	0.905
	BI2	0.902	67.434			
	BI3	0.929	94.892			
Perceived credibility (PCB)	PCB1	0.817	46.469	0.735	0.917	0.879
	PCB2	0.871	55.420			
	PCB3	0.889	66.637			
	PCB4	0.849	47.769			
Perceived convenience (PCV)	PCV1	0.908	87.463	0.767	0.908	0.848
	PCV2	0.863	52.135			
	PCV3	0.856	47.187			
Perceived ease of use (PEOU)	PEOU1	0.887	61.247	0.749	0.923	0.888
	PEOU2	0.845	49.959			
	PEOU3	0.867	54.512			
	PEOU4	0.864	47.859			
Perceived privacy risks (PPR)	PPR1	0.949	36.093	0.925	0.961	0.920
	PPR2	0.974	51.399			
Perceived behavioral control (PBC)	PBC1	0.914	86.912	0.747	0.855	0.671
	PBC2	0.812	20.231			
Perceived usefulness (PU)	PU1	0.874	57.958	0.757	0.926	0.893
	PU2	0.852	43.988			
	PU3	0.866	56.348			
	PU4	0.889	66.871			
Social influence (SI)	SI1	0.929	74.034	0.853	0.920	0.827
	SI2	0.918	67.289			
Perceived effective use (PEU)	PEU	1.000	-		1.000	1.000

**Table 5:** Square root of the AVE (in bold) and correlation coefficients between constructs.

	ATT	BI	PEOU	PCV	PVB	PEU	PPR	PBC	PU	SI
ATT	**0.892**									
BI	0.788	**0.917**								
PEOU	0.667	0.683	**0.866**							
PCV	0.749	0.692	0.695	**0.876**						
PCB	0.784	0.695	0.644	0.744	**0.857**					
PEU	0.743	0.783	0.636	0.674	0.655	**1**				
PPR	−0.192	−0.118	−0.139	−0.127	−0.228	−0.086	**0.961**			
PBC	0.728	0.662	0.63	0.703	0.751	0.611	−0.185	**0.864**		
PU	0.773	0.723	0.727	0.781	0.729	0.692	−0.221	0.736	**0.870**	
SI	0.354	0.298	0.313	0.309	0.377	0.247	−0.021	0.395	0.369	**0.923**

Note: ATT = Attitude; BI = Behavioral intention; PCB = Perceived credibility; PCV = Perceived convenience; PEOU = Perceived ease of use; PPR = Perceived privacy risk; PBC = Perceived behavioral control; PU = Perceived usefulness; SI = Social influence; PEU = Perceived effective use.

**Table 6:** Matrix of outer loadings and cross-loadings of the measurement items.

	ATT	BI	PCB	PCV	PEOU	PEU	PPR	PBC	PU	SI
ATT1	0.892	0.720	0.689	0.674	0.599	0.695	−0.130	0.647	0.665	0.312
ATT2	0.896	0.724	0.693	0.639	0.587	0.651	−0.183	0.654	0.705	0.314
ATT3	0.887	0.664	0.716	0.692	0.598	0.641	−0.201	0.645	0.698	0.322
BI1	0.727	0.919	0.656	0.647	0.652	0.722	−0.106	0.629	0.680	0.311
BI2	0.709	0.902	0.594	0.622	0.583	0.694	−0.084	0.543	0.630	0.246
BI3	0.732	0.929	0.658	0.633	0.642	0.735	−0.135	0.646	0.677	0.263
PCB1	0.685	0.609	0.817	0.716	0.580	0.598	−0.125	0.689	0.681	0.351
PCB2	0.668	0.600	0.871	0.620	0.575	0.539	−0.213	0.637	0.608	0.315
PCB3	0.707	0.647	0.889	0.648	0.588	0.599	−0.255	0.654	0.650	0.303
PCB4	0.619	0.512	0.849	0.548	0.447	0.496	−0.192	0.581	0.544	0.322
PCV1	0.696	0.654	0.709	0.908	0.653	0.632	−0.112	0.670	0.719	0.286
PCV2	0.602	0.559	0.618	0.863	0.565	0.519	−0.048	0.580	0.619	0.281
PCV3	0.666	0.600	0.624	0.856	0.604	0.615	−0.171	0.592	0.710	0.245
PEOU1	0.592	0.615	0.555	0.596	0.887	0.569	−0.122	0.572	0.627	0.263
PEOU2	0.557	0.567	0.562	0.595	0.845	0.550	−0.113	0.544	0.643	0.283
PEOU3	0.562	0.574	0.534	0.615	0.867	0.544	−0.104	0.519	0.609	0.260
PEOU4	0.598	0.608	0.577	0.600	0.864	0.537	−0.142	0.545	0.638	0.278
PEU	0.743	0.783	0.655	0.674	0.636	1.000	−0.086	0.611	0.692	0.247
PPR1	−0.155	−0.097	−0.192	−0.089	−0.103	−0.054	0.949	−0.139	−0.176	0.040
PPR2	−0.207	−0.126	−0.24	−0.146	−0.156	−0.104	0.974	−0.207	−0.24	−0.063
PBC1	0.713	0.662	0.711	0.682	0.625	0.606	−0.182	0.914	0.704	0.279
PBC2	0.521	0.456	0.574	0.513	0.440	0.427	−0.133	0.812	0.552	0.439
PU1	0.686	0.623	0.668	0.668	0.629	0.611	−0.218	0.672	0.874	0.355
PU2	0.629	0.598	0.588	0.699	0.650	0.549	−0.187	0.612	0.852	0.321
PU3	0.666	0.626	0.627	0.658	0.603	0.580	−0.183	0.609	0.866	0.312
PU4	0.708	0.669	0.654	0.696	0.649	0.664	−0.183	0.666	0.889	0.296
SI1	0.337	0.285	0.368	0.295	0.290	0.227	−0.036	0.369	0.326	0.929
SI2	0.317	0.265	0.328	0.275	0.288	0.229	−0.001	0.361	0.356	0.918

**Table 7:** Results of hypothesis testing using 5000 bootstrap subsamples.

Hypotheses		Path Coefficients	*t*-Value	*p* Value	Support? (Yes/No)
H1	BI → PEU	0.673	15.278	<0.001	Yes
H2	ATT → BI	0.534	10.564	<0.001	Yes
H3	PU → ATT	0.324	5.558	<0.001	Yes
H4	PU → BI	0.253	5.093	<0.001	Yes
H5	PEOU → PU	0.440	8.452	<0.001	Yes
H6	SI → ATT	0.027	0.877	0.381	No
H7	SI → BI	−0.026	0.743	0.458	No
H8	PBC → BI	0.109	2.019	0.043	Yes
H9	PBC → PEU	0.165	3.109	0.002	Yes
H10	PEOU → PCV	0.369	7.443	<0.001	Yes
H11	PCV → ATT	0.196	3.294	0.001	Yes
H12	PCB → PCV	0.507	10.444	<0.001	Yes
H13	PCB → PU	0.432	8.672	<0.001	Yes
H14	PCB → ATT	0.391	7.675	<0.001	Yes
H15	PPR → PU	−0.061	1.981	0.048	Yes
H16	PPR → ATT	−0.006	0.200	0.842	No
H17	PPR → BI	0.060	2.078	0.038	Yes

Note: ATT = Attitude; BI = Behavioral intention; PCB = Perceived credibility; PCV = Perceived convenience; PEOU = Perceived ease of use; PPR = Perceived privacy risks; PBC = Perceived behavioral control; PU = Perceived usefulness; SI = Social influence; PEU = Perceived effective use.

## Data Availability

The data presented in this study are available on request from the corresponding author. The data are not publicly available due to privacy issue.
